# Supplementary material for: Effects of In-Hospital Exercise on Frailty in Patients with Hepatocellular Carcinoma
Source: Cancers (Basel). 2021 Jan 7;13(2):194. doi: 10.3390/cancers13020194 (PMC7826707; doi:10.3390/cancers13020194)
Supplement: Supplementary file 1 [file cancers-13-00194-s001.pdf]

Supplementary Material: Effects of In-Hospital Exercise on Frailty in Patients with Hepatocellular Carcinoma

Jin Tsuchihashi, Shunji Koya, Keisuke Hirota, Noboru Koga, Hayato Narao, Manabu Tomita, Takumi Kawaguchi, Ryuki Hashida, Dan Nakano, Tsubasa Tsutsumi, Sachiyo Yoshio, Hiroo Matsuse, Taku Sanada, Kazuo Notsumata and Takuji Torimura

Table S1. Consensus on Exercise Reporting Template (CERT) for the exercise program of this study.

CERT Item No.	Stretching	Resistance Training	Balance Training	Aerobic Training
1. Equipment	No equipment	No equipment	Parallel bars or handrails were used for fall prevention.	Bicycle ergometer (Konami Sports Co., Ltd., Tokyo, Japan, or Senoh Corporation, Chiba, Japan) or recumbent cross trainer (NuStep®, Senoh Corporation) [6] was used.
2. Qualifications, expertise, and/or training	Six physical therapists delivered the exercise program. They were certified for the rehabilitation of cancer patients and had an average of 14.5 years (range 9–25 years) of clinical experience with HCC. Two (30%) of these physical therapists had a Doctor of Philosophy (Ph.D.) degree qualification.	Six physical therapists delivered the exercise program. They were certified for the rehabilitation of cancer patients and had an average of 14.5 years (range 9–25 years) of clinical experience with HCC. Two (30%) of these physical therapists had a Doctor of Philosophy (Ph.D.) degree qualification.	Six physical therapists delivered the exercise program. They were certified for the rehabilitation of cancer patients and had an average of 14.5 years (range 9–25 years) of clinical experience with HCC. Two (30%) of these physical therapists had a Doctor of Philosophy (Ph.D.) degree qualification.	Six physical therapists delivered the exercise program. They were certified for the rehabilitation of cancer patients and had an average of 14.5 years (range 9–25 years) of clinical experience with HCC. Two (30%) of these physical therapists had a Doctor of Philosophy (Ph.D.) degree qualification.
3. Individual/group	Individual	Individual	Individual	Individual
4. Supervised/unsupervised	Supervised	Supervised	Supervised	Supervised

Table 1. *Cont.*

CERT Item No.	Stretching	Resistance Training	Balance Training	Aerobic Training
5. Adherence	Adherence was defined by the percentage of supervised exercise sessions completed.	Adherence was defined by the percentage of supervised exercise sessions completed.	Adherence was defined by the percentage of supervised exercise sessions completed.	Adherence was defined by the percentage of supervised exercise sessions completed.
6. Motivation	The therapist gives comments for the patients' effort. The oral agreements between the patient and the therapist were reinforced in writing in the form of a "treatment contract".	The therapist gives comments for the patients' effort. The oral agreements between the patient and the therapist were reinforced in writing in the form of a "treatment contract".	The therapist gives comments for the patients' effort. The oral agreements between the patient and the therapist were reinforced in writing in the form of a "treatment contract".	The therapist gives comments for the patients' effort. The oral agreements between the patient and the therapist were reinforced in writing in the form of a "treatment contract".
7a. Progression	Each stretch was held for 10–20 s. Total stretching time was 3–5 min.	One set comprised of 10 repetitions, and a maximum of 3 sets was performed. Total resistance training time was 5–10 min.	The patient tried to keep position in a one-leg stance and tandem stance for 1 min. These were done once on each side. Total balance training time was 5 min.	The intensity of exercise was adjusted to maintain a subjective rating of perceived exertion of 11–13 points on the Borg scale Total aerobic training time was 10–15 min.
7b. A detailed description of how the exercise program was progressed	Patients progressed each stretches with a feeling of tightness and slight discomfort.	The resistance training used their own weight or own manual resistance.	The patient tried to keep position in a one-leg stance and tandem stance for up to 1 min.	The individual exercise intensity was adapted during each session by adjusting the load or the cycling speed. The exercise duration was gradually increased by 15 min.
8. Exercise description	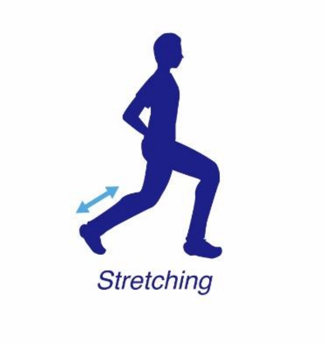	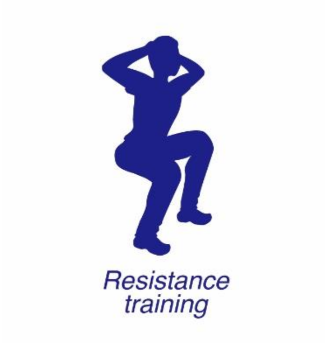	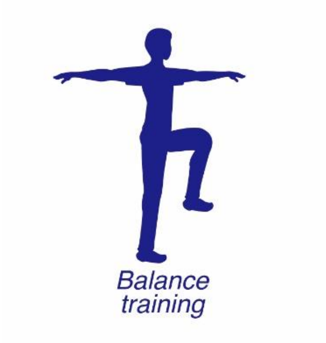	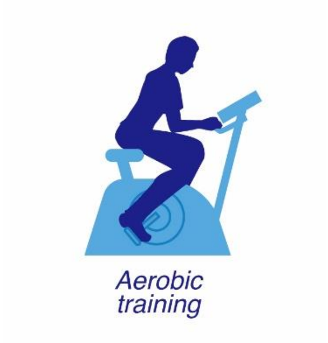

Table 1. *Cont.*

CERT Item No.	Stretching	Resistance Training	Balance Training	Aerobic Training
9. Home programme component	There was no other exercise and no functional tasks.	There was no other exercise and no functional tasks.	There was no other exercise and no functional tasks.	There was no other exercise and no functional tasks.
10. Non-exercise components	Chemotherapy for HCC and nutritional therapy including BCAA supplementation	Chemotherapy for HCC and nutritional therapy including BCAA supplementation	Chemotherapy for HCC and nutritional therapy including BCAA supplementation	Chemotherapy for HCC and nutritional therapy including BCAA supplementation
11. Adverse events	There was no severe adverse event.	There was no severe adverse event.	There was no severe adverse event.	There was no severe adverse event.
12. Setting	The exercise was performed in a rehabilitation room or hospital ward under the supervision of an experienced physical therapist.	The exercise was performed in a rehabilitation room or hospital ward under the supervision of an experienced physical therapist.	The exercise was performed in a rehabilitation room or hospital ward under the supervision of an experienced physical therapist.	The exercise was performed in a rehabilitation room or hospital ward under the supervision of an experienced physical therapist.
13. Details of the intervention	Each stretch was held for 10–20 s at the point of tightness or slight discomfort was felt. The physical therapist showed a role model of the stretch and checked the stretching form, tightness, and discomfort. The stretches targeted the muscles of the quadriceps femoris muscles, hamstrings, hip adductor muscles, gastrocnemius, back muscles, and shoulder muscles. Total stretching time was 3–5 min. The frequency of stretches was performed 5 times/week.	One set comprised ten repetitions, and a maximum of 3 sets was performed. Physical therapists adjusted the method of exercises and environment to be able to complete 10 repetitions for the exercise. The resistance training included the following four exercises: (1) hip hinge movement (good-morning exercise), (2) towel air pull-down, (3) squats, and (4) calf raises. Total resistance training time was 5–10 min. The frequency of resistance training was performed 5 times/week.	Balance training was performed in both a one-leg stance or tandem stance using parallel bars and handrails. A one-leg stance maintaining a horizontal position in a one-legged position. The tandem stance method maintains a posture while standing in a straight line. These were done once on each side. Total balance training time was 5 min. The frequency of balance training was performed 5 times/week.	Aerobic training was performed using a bicycle ergometer, a recumbent cross-trainer, or by walking. All aerobic training intensities were adjusted to the subjective rating of perceived exertion of 11–13 points on the Borg scale. Total aerobic training time was 10–15 min. The frequency of aerobic exercise was 5 times/week.
14a. Generic exercise/tailored exercise	Generic exercise	Generic exercise	Generic exercise	Tailored exercise

Table 1. *Cont.*

CERT Item No.	Stretching	Resistance Training	Balance Training	Aerobic Training
14b. Detail of tailored exercise	Not applicable	Not applicable	Not applicable	All aerobic training intensities were adjusted to the subjective rating of perceived exertion of 11–13 points on the Borg scale by adjusting the load or the cycling speed. The exercise duration was gradually increased up to 15 min.
15. Starting level	Each stretch was held for 10–20 s at the point of tightness or slight discomfort.	One set comprised of 10 repetitions, and a maximum of 3 sets was performed. Physical therapists adjusted the method of exercises and environment to be able to complete 10 repetitions for the exercise.	The patient tried to keep position in a one-leg stance and tandem stance for up to 1 min.	All aerobic training intensities were adjusted to the subjective rating of perceived exertion of 11–13 points on the Borg scale.
16a. Adherence or compliance	The physical therapist attended a 5 min session covering the delivery of the exercise program.	The physical therapist attended a 5 min session covering the delivery of the exercise program.	The physical therapist attended a 5 min session covering the delivery of the exercise program.	The physical therapist attended a 5 min session covering the delivery of the exercise program.
16b. The extent to which intervention was delivered as planned.	The median implementation rate was 100% (IQR: 75; 100). When patients had a fever (>38 °C), exercise was canceled.	The median implementation rate was 100% (IQR: 75; 100). When patients had a fever (>38 °C), exercise was canceled.	The median implementation rate was 100% (IQR: 75; 100). When patients had a fever (>38 °C), exercise was canceled.	The median implementation rate was 100% (IQR: 75; 100). When patients had a fever (>38 °C), exercise was canceled.

Abbreviations: CERT; Consensus on Exercise Reporting Template, HCC; hepatocellular carcinoma; BCAA; branched-chain amino acids, IQR; interquartile range.
